# First report of *Borrelia burgdorferi* sensu stricto detection in a commune genospecies in *Apodemus agrarius* in Gwangju, South Korea

**DOI:** 10.1038/s41598-021-97411-3

**Published:** 2021-09-14

**Authors:** Choon Mee Kim, So Young Park, Dong-Min Kim, Jung Wook Park, Jae Keun Chung

**Affiliations:** 1 Premedical Science, College of Medicine, Chosun University, Gwangju, South Korea; 2 Department of Internal Medicine, College of Medicine, Chosun University, 588 Seosuk-dong, Dong-gu, Gwangju, 501-717 Republic of Korea; 3 Division of Infectious Disease Investigation, Health and Environment Research Institute of Gwangju City, Gwangju, South Korea

**Keywords:** Microbiology, Diseases, Molecular medicine

## Abstract

Lyme disease is a tick-borne infectious disease caused by the *Borrelia burgdorferi* sensu lato complex. However, the distribution of *Borrelia* genospecies and the tissue detection rate of *Borrelia* in wild rodents have rarely been investigated. Here, we studied 27 wild rodents (*Apodemus agrarius*) captured in October and November 2016 in Gwangju, South Korea, and performed nested polymerase chain reaction targeting *pyrG* and *ospA* to confirm *Borrelia* infection. Eight rodents (29.6%) tested positive for *Borrelia* infection. The heart showed the highest infection rate (7/27; 25.9%), followed by the spleen (4/27; 14.8%), kidney (2/27; 7.4%), and lungs (1/27; 3.7%). The *B. afzelii* infection rate was 25.9%, with the highest rate observed in the heart (7/27; 25.9%), followed by that in the kidney and spleen (both 2/27; 7.4%). *B. garinii* and *B. burgdorferi* sensu stricto were detected only in the spleen (1/27; 3.7%). This is the first report of *B. burgdorferi* sensu stricto infection in wild rodents in South Korea. The rodent hearts showed a high *B. afzelii* infection rate, whereas the rodent spleens showed high *B. garinii* and *B. burgdorferi* sensu stricto infection rates. Besides *B. garinii* and *B. afzelii*, *B. burgdorferi* sensu stricto may cause Lyme disease in South Korea.

## Introduction

Lyme borreliosis is a vector-borne disease characterized by polymorphic clinical manifestations (cutaneous, rheumatological, and neurological) that is mostly reported in North America, Europe, and Asia. The spirochetes that cause Lyme borreliosis can spread to other tissues and organs and cause serious multisystem infection involving the skin, nervous system, joints, or heart^1–4^. In the United States, changes in land use practices and a marked increase in the deer population have increased the risk of exposure to ticks infected with Lyme disease-causing spirochetes^5^. James et al. reported the need for environmental studies on tick abundance and *Borrelia burgdorferi* sensu lato infection in ticks for reducing exposure risk and predicting future trends in pathogen prevalence and distribution patterns in response to environmental changes^6^.

The *Borrelia burgdorferi* sensu lato species complex (comprising *Borrelia burgdorferi* sensu stricto, *B. afzelii*, and *B. garinii*, among others) causes Lyme disease. The genospecies of this complex are transmitted by different species of ticks (e.g., *Ixodes scapularis* and *I. persulcatus*) and are responsible for causing Lyme borreliosis in humans in different geographical regions^4,7,8^. *B. burgdorferi* sensu stricto is known to cause Lyme disease in North America, and, less extensively, in Europe. At least five *Borrelia* species (*B. afzelii*, *B. garinii*, *B. burgdorferi* sensu stricto, *B. spielmanii*, and *B. bavariensis*) have been identified that cause Lyme disease in Europe. *B. afzelii* and *B. garinii* are the predominant species in Europe, whereas *B. garinii* is predominant in Asia. *B. burgdorferi* sensu stricto is the only species known to cause Lyme borreliosis in northern America, but has rarely been detected in Asia^3,4^. *B. burgdorferi* sensu stricto was isolated for the first time from a human skin biopsy sample in Taiwan^9^. However, to our knowledge, *B. burgdorferi* sensu stricto has not been isolated in Japan or South Korea to date^10^.

In South Korea, *B. burgdorferi* sensu lato was first isolated from *Ixodes* ticks and the rodent species *Apodemus agrarius* in 1992^11^. In 2002, nine *B. afzelii* strains were isolated from *Ixodes nipponensis* and *A. agrarius* in Chungju, South Korea, and their heterogeneous characteristics, which were different from those of previously reported *B. afzelii* strains, were identified using polymerase chain reaction-restriction fragment length polymorphism (PCR-RFLP) of the *ospC* gene and *rrf *(*5S*)*-rrl *(*23S*) intergenic space^12^. Moreover, in 2020, the prevalence and distribution of five *B. burgdorferi* sensu lato genospecies (*B. afzelii*, *B. valaisiana*, *B. yangtzensis, B. garinii*, and *B. tanukii*) were reported based on the results of nested PCR targeting partial flagellin B gene sequences and sequencing in ticks isolated from wild rodents in South Korea^13^.

Lyme disease is a Group 3 infectious disease in South Korea, and the number of cases reported to the Korea Disease Control and Prevention Agency has gradually increased from 2011 to 2020, with an average of 15.4 cases reported per year, and 31, 23, 23, and 12 cases reported each year from 2017 to 2020, respectively^14^.

The diagnosis of Lyme disease is confirmed based on positive results in an indirect immunofluorescence assay or enzyme-linked immunosorbent assay via western blot analysis or by isolating and identifying the pathogen from clinical specimens from patients, including blood samples. In addition, the molecular techniques used for classifying and identifying *Borrelia* spp. and *B. burgdorferi* include PCR targeting rRNA genes, *flaB*, *recA*, *p66*, and the plasmid-encoded gene *ospA*; DNA-DNA homology analysis, ribotyping, PCR-RFLP analysis, pulsed-field gel electrophoresis, randomly amplified polymorphic DNA fingerprinting, multilocus sequence typing/multilocus sequence analysis, and whole genome sequencing^2,15–21^. The process of culturing clinical specimens to detect *B. burgdorferi* is labor-intensive, expensive, and applicable only to untreated patients, and therefore, is not used in clinical practice. However, microorganisms can be directly detected in clinical specimens using PCR, and their genotype can be confirmed through sequencing without isolating the pathogens^17^. Nested PCR is known to exhibit a sensitivity 100 times greater than that of conventional PCR; hence, nested PCR can be used to increase the diagnostic sensitivity for Lyme disease^17,22^. Nested PCR targeting the *rrf* (5S)-*rrl* (23S) intergenic spacer and *ospA* (encoding the outer surface protein A) gene or the 16S rRNA and *pyrG* (encoding CTP synthase) genes was performed, along with sequence analysis, to detect *Borrelia* DNA in clinical samples^17,20^.

In this study, we investigated the infection rate in 27 wild rodents (*A. agrarius*) captured in October and November 2016 using nested PCR targeting the *Borrelia*-specific genes *pyrG* and *ospA* and direct DNA sequencing with rodent tissue samples. The distribution and infection rate of *Borrelia* genospecies in wild rodents, which serve as reservoirs for the pathogens of tick-borne infectious disease, have rarely been investigated. In addition, we reported the rates of *Borrelia* infection in the different organs of the wild rodents and investigated the differences in the organ-specific detection rate of each *Borrelia* species.

## Results

### PCR and tissue detection rates of *Borrelia* species in captured wild rodents

Twenty-seven wild rodents were captured using Sherman live traps during October and November 2016 in two regions of Gwangju City in South Korea. All captured rodents were identified as *A. agrarius*. *Borrelia*-specific *pyrG* and *ospA* nested PCR revealed that 8 of the 27 rodents were infected with the pathogens in the spleen, kidney, lungs, and heart (Table 1).

**Table 1:** PCR detection of *Borrelia* species in the organs of wild mice^†^.

No. of captured mice	Lung		Spleen		Heart		Kidney	
	*pyrG* N-PCR	*ospA* N-PCR	*pyrG* N-PCR	*ospA* N-PCR	*pyrG* N-PCR	*ospA* N-PCR	*pyrG* N-PCR	*ospA* N-PCR
10-1	−	−	−	−	−	−	−	−
10-2	−	−	−	−	−	−	−	−
10-3	−	−	−	−	+ (*B. afzelii*)	+ (*B. afzelii*)	−	−
10-4	−	−	−	−	−	−	−	−
10-5	−	−	−	−	+ (*B. afzelii*)	+ (*B. afzelii*)		−
10-6	−	−	−	−	−	−	−	−
10-7	−	−	−	−	−	−	−	−
10-8	−	−	+ (*B. burgdorferi*)	+ (*B. burgdorferi*)	−	−	−	−
10-10	−	−	−	−	−	−	−	−
10-11	+ (*B. afzelii*)	+ (*B. afzelii*)	+ (*B. garinii*)	−	+ (*B. afzelii*)	+ (*B. afzelii*)	+ (*B. afzelii*)	−
10-12	−	−	−	−	−	−	−	−
10-13	−	−	−	−	−	−	−	−
11-1	−	−	−	−	+ (*B. afzelii*)	+ (*B. afzelii*)	+ (*B. afzelii*)	+ (*B. afzelii*)
11-2	−	−	−	−	−	−	−	−
11-3	−	−	−	−	−	−	−	−
11-4	−	−	−	−	−	−		−
11-5	−	−	−	−	−	−	−	−
11-6	−	−	+ (*B. afzelii*)	−	+ (*B. afzelii*)	+ (*B. afzelii*)		−
11-7	−	−	−	−	−	−	−	−
11-8	−	−	−	−	−	−		−
11-9	−	−	−	−	−	−	−	−
11-10	−	−	−	−	−	−	−	−
11-11	−	−	−	−	−	−	−	−
11-12	−	−	−	−	−	−	−	−
11-13	−	−	+ (*B. afzelii*)	−	+ (*B. afzelii*)	−	−	−
11-14	−	−	−	−	−	−	−	−
11-15	−	−	−	+ (*B. afzelii*)	+ (*B. afzelii*)	+ (*B. afzelii*)	−	−

^†^*pyrG*, CTP synthase gene; *ospA*, outer surface protein A gene; N-PCR, nested PCR; −, negative; +, positive.

In *pyrG* nested PCR, the overall rate of positive response to *Borrelia* species was 29.6% (8/27). Among the studied organs, the detection rate was the highest in the heart (25.9%, 7/27). The kidney and spleen showed positive detection rates of 7.4% (2/27) and 14.8% (4/27), respectively. The *B. afzelii* infection rate was 25.9% (7/27) in the wild rodents. The heart showed the highest positive detection rate (25.9%, 7/27), and the kidney and spleen had a positive detection rate of 7.4% (2/27), respectively. The infection rate for both *B. garinii* and *B. burgdorferi* sensu stricto was 3.7% (1/27), and both bacteria were detected only in the spleens.

In *ospA* nested PCR, the heart tissues from 6 of 27 wild rodents exhibited a positive response to *B. afzelii*, with an infection rate of 22.2%. The infection rate was 3.7% (1/27) in the spleen, kidney, and lung tissues. In a wild rodent that showed a *Borrelia*-positive result in *pyrG* nested PCR (Chosun M10-8 Sp, which indicates the spleen of wild rodent no. 8 captured in October 2016), *B. burgdorferi* sensu stricto was also detected by *ospA* nested PCR in the spleen.

Using *pyrG* nested PCR, the *B. afzelii* infection rate was found to be 25% (3/12) in October and 26.7% (4/15) in November, and the hearts of the animals showed the highest infection rate in both months. With respect to *B. garinii*, a positive response was detected in the spleen of only one animal captured in October (Chosun M10-11 Sp). This animal exhibited co-infection with *B. garinii* (detected in the spleen) and *B. afzelii* (detected in the heart, kidney, and lungs). Additionally, infection by *B. burgdorferi* sensu stricto in the spleen was confirmed in only one wild rodent captured in October (Chosun M10-8 Sp).

### Sequence analysis and phylogenetic analysis

**Figure 1:**
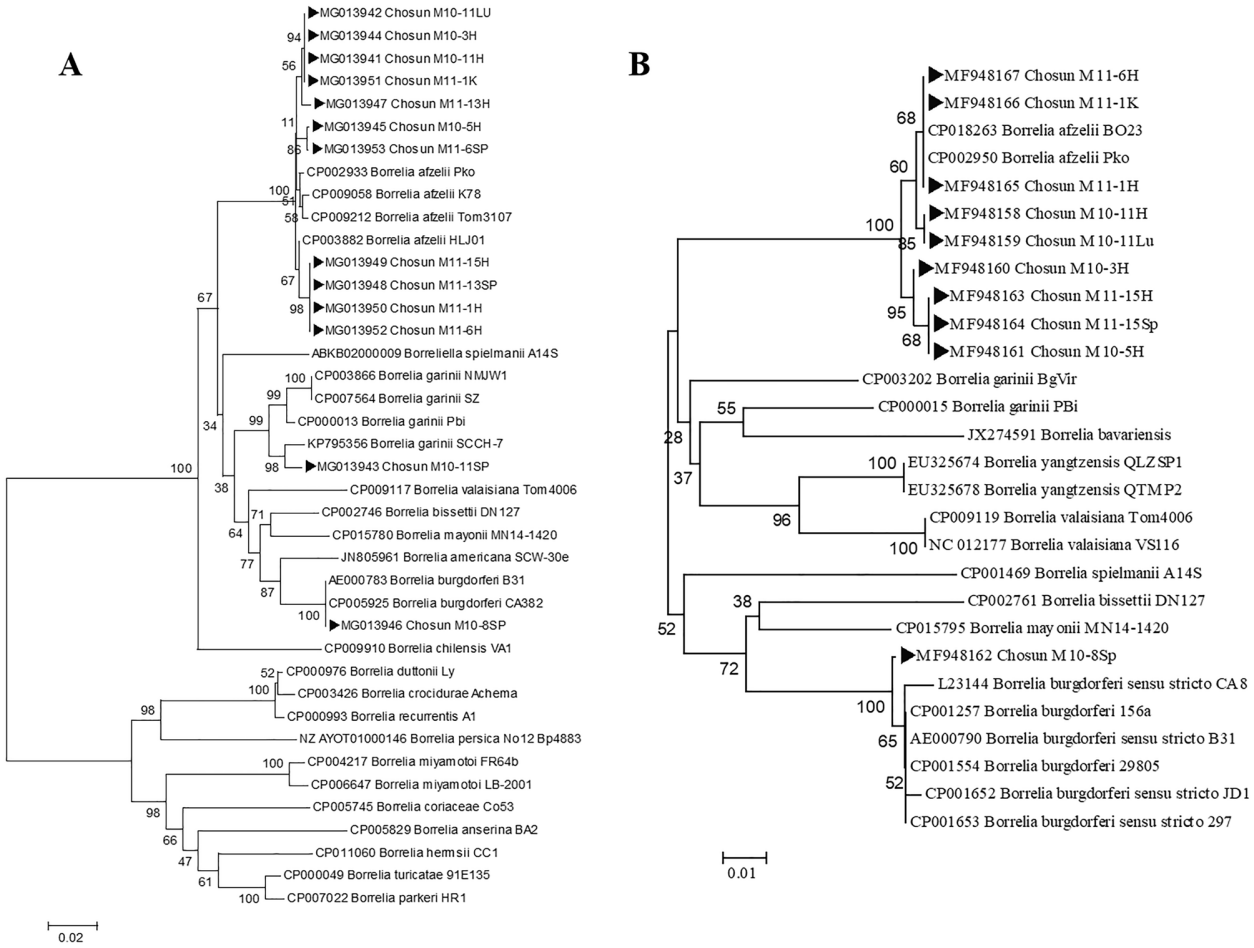
A phylogenetic tree constructed using the *pyrG* (**A**, 675 bp) and *ospA* (**B**, 285 bp) sequences retrieved from GenBank and obtained from the tissue DNA of the wild rodents captured (▶). The scale bars indicate 0.02 (**A**) or 0.01 (**B**) base substitutions per site. The GenBank accession numbers are shown in the tree.

A phylogenetic tree was constructed based on the partial nucleotide sequences of *pyrG* (675 bp) and *ospA* (285 bp) segments obtained from *Borrelia-*positive tissue specimens and various *Borrelia* strains, such as *B. afzelii* HLJ01, *B. garinii* SCCH-7, and *B. burgdorferi* B31, among others, from GenBank. The phylogenetic trees generated using the *pyrG* and *ospA* gene sequences exhibited similar topologies. All *pyrG* sequences obtained from the heart, kidneys, spleen, and lungs of the wild rodents (M10-3, M10-5, M10-11, M11-1, M11-6, M11-13, and M11-15) clustered with *B. afzelii*. Additionally, the *pyrG* sequences from the spleen of M10-11 (Chosun M10-11Sp) formed a cluster with *B. garinii*, whereas the *pyrG* sequences from the spleen of another wild rodent (Chosun M10-8 Sp) formed a cluster with *B. burgdorferi* sensu stricto (Fig. 1).

We evaluated the similarities between *Borrelia pyrG* (675 bp) and *ospA* (285 bp) nucleotide sequences obtained from GenBank and *Borrelia-*positive tissue specimens using LaserGene v6 (DNASTAR, Madison, WI, USA). Sequence similarity comparison showed that the *pyrG* sequences obtained from the organs of wild rodents (Chosun M10-3H, M10-5H, M10-11H and 10-11Lu, M11-1H and 11-1K, M11-6H and 11-6Sp, M11-13H and 11-13Sp, and M11-15H) exhibited more than 99% similarity with the *pyrG* sequences from *B. afzelii* HLJ01 strains isolated from Chinese patients (GenBank accession number CP003882). Moreover, *B. garinii* DNA isolated from the spleen of M10-11 (Chosun M10-11 Sp) exhibited more than 98.4% homology with the *pyrG* gene sequence of *B. garinii* strain SCCH-7 isolated from rodents in the USA (GenBank accession number KP795356). Lastly, the *pyrG* DNA of *B. burgdorferi* sensu stricto isolated from the spleen of M10-8 (Chosun M10-8 Sp) showed 100% and 99.4% similarity to that of *B. burgdorferi* sensu stricto B31_NRZ strain isolated from *I. scapularis* (GenBank accession number AE000783) and of *B. burgdorferi* sensu stricto 297 strain isolated from human cerebrospinal fluid in the USA (GenBank accession number KF170281), respectively (Table 2).

**Table 2:** Similarity between *Borrelia pyrG* gene sequences from GenBank and the wild rodents captured in this study^†^.

Genospecies of *Borrelia*	Strains	Origin of *Borrelia* strain		*pyrG* accession number	M10-3H	M10-5H	M10-8Sp	M10-11H	M10-11Lu	M10-11Sp	M11-1H	M11-1K	M11-6H	M11-6Sp	M11-13H	M11-13Sp	M11-15H
		Biological	Geographic														
*B. burgdorferi* sensu stricto	B31	*Ixodes scapularis*	USA	AE000783	91.9	91.9	100	91.9	91.9	92.4	92	91.9	92	92.1	91.7	92	92
	297	Human cerebrospinal fluid	USA	KF170281	91.4	91.6	99.4	91.4	91.4	91.8	91.6	91.4	91.6	91.7	91.3	91.6	91.6
*B. garinii*	20047	*I. ricinus*	France	CP028861	92.9	92.6	93.2	92.9	92.9	98.4	92.9	92.9	92.9	92.7	92.6	92.9	92.9
	NP81	*I. persulcatus*	Japan	AB555842	83	83.9	83.3	83	83	88.4	83	83	83	82.8	82.7	83	83
	SCCH-7	*Peromyscus gossypinus* (cotton mouse)	USA	KP795356	92.9	92.6	93.2	92.9	92.9	98.4	92.9	92.9	92.9	92.7	92.6	92.9	92.9
*B. afzelii*	BO23	Human skin	Sweden	CP018262	99.1	98.9	92.1	99.1	99.1	93.3	99	99.1	99	98.8	99	99	99
	HLJ01	Human	China	CP003882	99.4	99.2	92.1	99.4	99.4	93.2	99.6	99.4	99.6	99.4	99.6	99.6	99.6
*B. bissettii*	DN127	*I. pacificus*	USA	CP002746	92.6	92	94.4	92.6	92.6	93.5	92.6	92.6	92.6	92.1	92.9	92.6	92.6
*B. spielmanii*	A14S	Human skin	Netherlands	ABKB02000009	92.9	92.6	93.3	92.9	92.9	92.1	92.6	92.9	92.6	92.6	92.6	92.6	92.6
*B. valaisiana*	VS116	*I. ricinus*	Switzerland	ABCY02000001	91.3	91.3	93.5	91.3	91.3	92.9	91.1	91.3	91.1	91.3	90.8	91.1	91.1
*B. sinica*	CMN3	*Niviventer* sp.	China	AB526135	82.5	83.5	81.8	82.5	82.5	82.6	82.4	82.5	82.4	82.2	82.1	82.4	82.4

^†^*pyrG*, CTP synthase gene.

The nucleotide sequence comparison of *ospA* from Chosun M10-8 Sp infected with *B. burgdorferi* sensu stricto strains revealed a high variability in the degree of sequence similarity, ranging from 73.7 to 99.6%. The *ospA* sequences of *B. burgdorferi* sensu stricto isolated from Chosun M10-8 Sp exhibited 99.6% similarity with those of *B. burgdorferi* sensu stricto B31 strain (GenBank accession number AE000790) and *B. burgdorferi* sensu stricto 297 strain isolated in the USA (GenBank accession number CP001653) (Table 3).

**Table 3:** Similarity between *Borrelia ospA* gene sequences from GenBank and wild rodents captured in this study^†^.

Genospecies of *Borrelia*	Strains	Origin of *Borrelia* strain		*ospA* accession number	M10-3H	M10-5H	M10-8Sp	M10-11H	M10-11Lu	M11-1H	M11-1 K	M11-6H	M11-15H	M11-15Sp
		Biological	Geographic											
*B. burgdorferi* sensu stricto	B31	*Ixodes scapularis*	USA	AE000790	89.1	88.8	99.6	88.8	88.8	88.4	88.4	88.4	88.8	88.8
	TWKM5	*Rattus norvegicus*	Taiwan	AF369941	64.6	64.2	73.7	64.6	64.6	64.2	64.2	64.2	64.2	64.2
	IP1	Human cerebrospinal fluid	France	DQ111052	74.4	74	83.2	74	74	73.7	73.7	73.7	74	74
	Sh-2-82	*I. dammini*	USA	DQ393311	74.4	74	83.2	74	74	73.7	73.7	73.7	74	74
	297	Human CSF	USA	CP001653	89.1	88.8	99.6	88.8	88.8	88.4	88.4	88.4	88.8	88.8
*B. garinii*	20047	*I. ricinus*	France	CP028862	90.9	90.5	89.1	91.9	91.9	91.6	91.6	91.6	90.5	90.5
*B. afzelii*	VS461	*I. ricinus*	Switzerland	Z29087	99.6	99.3	89.1	99.3	99.3	99.6	99.6	99.6	99.3	99.3
	BO23	Human skin	Sweden	CP018263	99.3	98.9	88.8	99.6	99.6	100	100	100	98.9	98.9
	J1	*I. persulcatus*	Japan	KM069290	84.9	84.6	75.4	84.6	84.6	84.9	84.9	84.9	84.6	84.6
*B. bissettii*	DN127	*I. pacificus*	USA	CP002761	87.4	87	90.9	87.7	87.7	87.4	87.4	87.4	87	87
*B. spielmanii*	A14S	Human skin	Netherlands	CP001469	87.7	87.4	88.1	87.7	87.7	87.4	87.4	87.4	87.4	87.4
*B. valaisiana*	VS116	*I. ricinus*	Switzerland	NC_012177	88.1	87.7	88.8	87.7	87.7	87.4	87.4	87.4	87.7	87.7

^†^*ospA*, outer surface protein A.

## Discussion

Lyme disease is a zoonotic disease transmitted by ticks and caused by the *B. burgdorferi* sensu lato complex, a group comprising approximately 20 species. *B. burgdorferi* sensu stricto, *B. afzelii*, *B. garinii*, *B. valaisiana*, and *B. lusitaniae* have been reported to cause the disease^23^.

In this study, we performed nested PCR targeting the *pyrG* and *ospA* genes of *Borrelia* species and detected infection by *Borrelia* genospecies, including *B. afzelii*, *B. garinii*, and *B. burgdorferi* sensu stricto, in *A. agrarius*, with a 29.6% (8/27) positive detection rate for *Borrelia* species.

In a study conducted in 2008, conventional PCR targeting *ospC*, a gene specific to *Borrelia,* was performed using genomic DNA extracted from 1618 ticks (420 pools) and 369 rodents (*A. agrarius*) captured close to the demilitarized zone of Gyeonggi Province, South Korea. Contrary to our results, the positive rate for *B. burgdorferi* sensu lato infection was found to be 1% (16/420) in ticks; however, the *Borrelia* infection rate in rodents was not reported^24^. We attempted to retrieve additional published reports on the detection rate of *Borrelia* genospecies in wild rodents, including *A. agrarius*, in South Korea; however, we did not find such reports. Recently, the *B. afzelii* detection rate was reported in ticks parasitizing domestic and wild animals in South Korea, including ticks from mammals (1.8%), horses (1.4%), wild boar (5.3%), native Korean goats (5.9%), and Korean water deer (0.8%), based on a nested PCR experiment targeting the 5S–23S rRNA of *Borrelia*^25^.

*Borrelia* species are transmitted primarily by *Ixodes* species, including *I. ricinus* and *I. persulcatus* in Europe, *I. scapularis* in North America, *I. nipponensis* in Japan, and *I. persulcatus* in China. Furthermore, in South Korea, *I. persulcatus*, *I. nipponensis*, *I. granulatus*, and *I. ovatus* have been reported as competent vectors of *Borrelia*^23,26^. In South Korea, *B. burgdorferi* sensu lato was isolated from *Ixodes* ticks and *A. agrarius* in 1993, and *B. afzelii* was isolated from *I. nipponensis* and *A. agrarius* in 2002^11,12^. Recently, *B. garinii* strain 935T was isolated from *I. persulcatus* ticks in South Korea. However, besides a single report on whole-genome sequencing, no other study has reported the detection of this organism in wild rodents or ticks^23^.

In the present study, a high positive detection rate for *B. afzelii* (25.9%) was found in specimens obtained from captured wild rodents, and the highest rate was observed in the heart tissues. While *B. garinii* and *B. burgdorferi* sensu stricto infection was also detected (3.7%), these bacteria only infected the spleen. In 2020, Cadavid et al. reported that when immunosuppressed adult *Macaca mulatta* were inoculated with *B. burgdorferi*, *B. burgdorferi* exhibited tropism for the meninges in the central nervous system and for connective tissues. Additionally, significant inflammation was noted only in the heart, and immunosuppressed animals inoculated with *B. burgdorferi* exhibited cardiac fiber degeneration and necrosis^27^. In 2017, Grillon et al., reported that *B. burgdorferi* sensu stricto and *B. afzelii* target the skin of mice regardless of the route of inoculation and cause persistent skin infection^28^. These results differ from ours, probably because we detected *Borrelia* spirochetes from each organ of the captured wild rodents, whereas Grillon et al. detected *Borrelia* from each organ after inoculating a susceptible animal.

Lyme disease spirochetes exhibit strain- and species-specific differences in tissue tropism. For example, infection by *B. burgdorferi* sensu stricto, *B. garinii*, and *B. afzelii*, the three major spirochetes causing Lyme disease, is characterized by distinct but overlapping clinical signs. Infection by *B. burgdorferi* sensu stricto, the most common causative agent of Lyme disease in the USA, is closely associated with arthritis, whereas that by *B. garinii* is related to neuroborreliosis, and that by *B. afzelii* is related to acrodermatitis (a type of chronic skin lesion). *B. burgdorferi* and *B. garinii* isolates were shown to cause severe arthritis in immunocompromised mice in animal studies^29,30^. The correlation between pancarditis and the marked tropism of *B. burgdorferi* in cardiac tissues was also reported in studies involving the autopsy of patients with sudden cardiac deaths associated with Lyme carditis^31^.

Lyme borreliosis spirochetes exhibit a high detection rate in a specific organ depending on the species, which could suggest a certain preference for the organ. These findings suggest that Lyme borreliosis spirochetes infecting rodents can be detected in the heart and spleen tissues, as *B. afzelii* exhibit a high detection rate in rodent hearts, whereas *B. garinii* and *B. burgdorferi* exhibit high detection rates in rodent spleens. According to Matuschka et al., infected Norway rats that served as reservoirs for Lyme disease spirochetes increased the infection risk for visitors to a city park in central Europe^32^. Based on the relatively high rate of *Borrelia* infection (29.6%) in the captured rodents in this study, the risk of Lyme disease in the Gwangju city, South Korea is predicted to be high. Therefore, additional research should be conducted to study the causative agents of Lyme disease in South Korea and further elucidate their prevalence and tissue tropism.

In conclusion, this is the first study to show the presence of *B. burgdorferi* sensu stricto in rodents captured in South Korea. *B. afzelii*, one of the causative agents of Lyme disease, exhibited a high positive detection rate (25.9%) in wild rodents, specifically in the heart tissues, captured in the areas around a metropolitan city in the southwestern region of South Korea, whereas *B. garinii* and *B. burgdorferi* exhibited high detection rates in the spleen.

Our findings suggest that along with *B. garinii* and *B. afzelii*, *B. burgdorferi* sensu stricto may also act as a causative agent of Lyme disease in South Korea, and different *Borrelia* species exhibit different tissue detection rates.

## Methods

### Study site and rodent capture

Wild rodents were captured using Sherman live traps (3″ × 3.5″ × 9″, USA) in two regions of Gwangsan-gu (35°09′19.2″ N, 126°45′05.4″ E) and Buk-gu (35°13′51.7″ N, 126°54′23.8″ E) in Gwangju Metropolitan City, South Korea, in October and November 2016^33^. The two regions in which the mouse traps were placed are located on a boundary that divides the urban and rural areas, and there were five types of locations (fallow ground, a ridge between rice fields, a boundary between a forest and field, area surrounding tombs, and area surrounding water) selected in both regions. For capturing wild rodents, 10 Sherman live traps were placed on a deserted area at each location once a month in October and November. Peanut butter-coated biscuits were used as the bait for the wild rodents, and the traps were set at approximately 10 a.m. and removed at approximately 8 p.m. Twenty-seven rodents were captured (twelve in October and fifteen in November). After capture, the animals were euthanized via inhalation of 5% isoflurane in accordance with an approved animal use protocol, and the spleen, kidneys, lungs, and heart were harvested and stored at − 20 °C^33^.

### DNA isolation

Approximately 25 mg of tissue specimens collected from the wild rodents were ground using a cell strainer (70 μm; Falcon, Corning, NY, USA) and 180 μL of ATL buffer from the QIAamp DNA Blood and Tissue Mini Kit (Qiagen, Hilden, Germany). The tissue suspension was treated with 20 µL of proteinase K and incubated overnight at 56 °C for complete tissue lysis. Genomic DNA was extracted using the QIAamp DNA Blood and Tissue Mini Kit (Qiagen) according to the manufacturer’s instructions.

### PCR amplification

To detect *Borrelia* DNA, nested PCR targeting the *pyrG* and *ospA* genes of *Borrelia* species was performed using genomic DNA extracted from the tissue specimens. For *pyrG* nested PCR, *pyrG*-1F/*pyrG*-1R primers (for the initial PCR step) and *pyrG*-2F/*pyrG*-2R primers (for nested PCR) were used^20^. For *ospA* nested PCR, Borrel-*ospA*F1/Borrel-*ospA*R1 primers (for the initial PCR step) and Borrel-*ospA*F2/Borrel-*ospA*R2 primers (for nested PCR) were used^17^. The primer sequences are listed in Table 4. PCR was performed using the AmpliTaq Gold 360 Master Mix (Applied Biosystems, Foster City, CA, USA) and an Applied Biosystems Veriti 96-Well Thermal Cycler. An enzyme reaction solution of 20 µL was used in the primary PCR; this solution was composed of 1 μL each of the forward and reverse primers (5 μM), 10 μL of Master Mix, 2 μL of a GC enhancer, and 4 μL of distilled water. Nested PCR was performed with the same reaction solution used in the initial PCR step, using the initial PCR product as the template. PCR was performed using gene-specific PCR primers at specific annealing temperatures under the following cycling conditions: 10 min at 94 °C for the pre-denaturation step, 30 cycles of 20 s at 94 °C, 30 s at the different annealing temperatures, 30 s–1 min at 72 °C, and a final extension step of 7 min at 72 °C. In each PCR run, a negative control (reaction mixture without the template DNA) was included. The genomic DNA of *B. burgdorferi* B31 Clone 5A1 was used as the positive control. The annealing temperatures are listed in Table 4. Upon the completion of PCR, the products were separated by electrophoresis on 1.2% agarose gels containing ethidium bromide.

**Table 4:** Oligonucleotide primers and PCR conditions used in this study^†^.

PCR	Primer name (sequence)	Annealing temperature (°C)	Product size (bp)	References
CTP synthase gene (*pyrG*) (external primer)	*pyrG*-1F (5′-ATTGCAAGTTCTGAGAATA-3′)	45	801	^20^
	*pyrG* -1R (5′-CAAACATTACGAGCAAATTC-3′)			
CTP synthase gene (*pyrG*) (internal primer)	*pyrG*-2F (5-′GATATGGAAAATATTTTATTTATTG-3′)	49	707	^20^
	*pyrG*-2R (5′-AAACCAAGACAAATTCCAAG-3′)			
	Borrel-*ospA*F1 (5′-GGGAATAGGTCTAATATTAGC-3′)	52	427	^17^
Outer surface protein A (*ospA*) (external primer)	Borrel-*ospA*R1 (5′-CTGTGTATTCAAGTCTGGTTCC-3′)			
Outer surface protein A (*ospA*) (internal primer)	Borrel-*ospA*F2 (5′-CAAAATGTTAGTAGCCTTGAT-3′)	52	314	^17^
	Borrel-*ospA*R2 (5′-TCTGTTGATGACTTGTCTTT-3′)			

^†^bp, base pair; PCR, polymerase chain reaction.

### Nucleotide sequencing

A QIAquick Gel Extraction Kit (Qiagen) was used to purify the PCR products, which were directly sequenced using the PCR primers and an automated sequencer (ABI Prism 3730XL DNA analyzer; Applied Biosystems) at Solgent (Deajeon, South Korea). To identify the bacteria, the sequences were analyzed using the BLAST network service (Ver 2.33; http://www.technelysium.com.au/chromas.html) available from the National Center for Biotechnology Information (National Institutes of Health, Rockville, MD, USA).

### Sequence similarity and phylogenetic analyses

The DNA sequence identity, contig generation, and homology comparison were confirmed using Lasergene v6 (DNASTAR, Madison, WI, USA) and the NCBI BlastN network service. After the sequences were concatenated, LaserGene v6 (DNASTAR) was used for sequence alignment and homology comparison.

A phylogenetic tree was constructed based on the partial sequences of *pyrG* (675 bp) and *ospA* (285 bp) obtained from the organ tissues of the wild rodents and from various *Borrelia* strains (listed in GenBank) using the neighbor joining method. ClustalX (version 2.0; http://www.clustal.org/) and Tree Explorer (DNASTAR) were used to construct the phylogenetic tree. To increase the reliability of the tree, bootstrap analysis was conducted with 1000 replicates. The sequence data generated in this study were submitted to NCBI GenBank (accession numbers MG013941 to MG013953 and MF948158 to MF948167), and the reference sequences were retrieved from the NCBI GenBank database.

### Ethics statement

This study was approved by the institutional review board of Chosun University. All rodents were euthanized in accordance with an animal use protocol approved by the Chosun University Institutional Animal Care and Use Committee (CIACUC) under the approval number CIACUC2016-A0003. The study was conducted in compliance with the ARRIVE guidelines for the reporting of animal studies.

## Data Availability

Data and materials are available upon request to the corresponding author.
